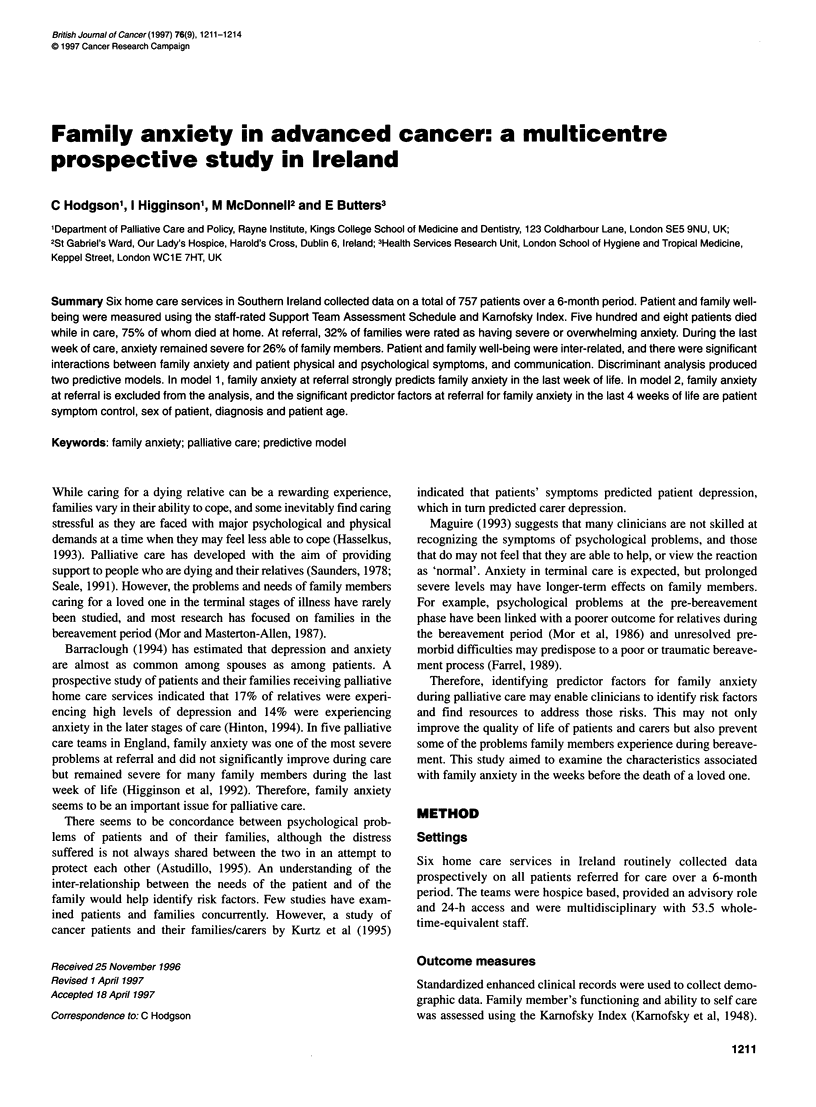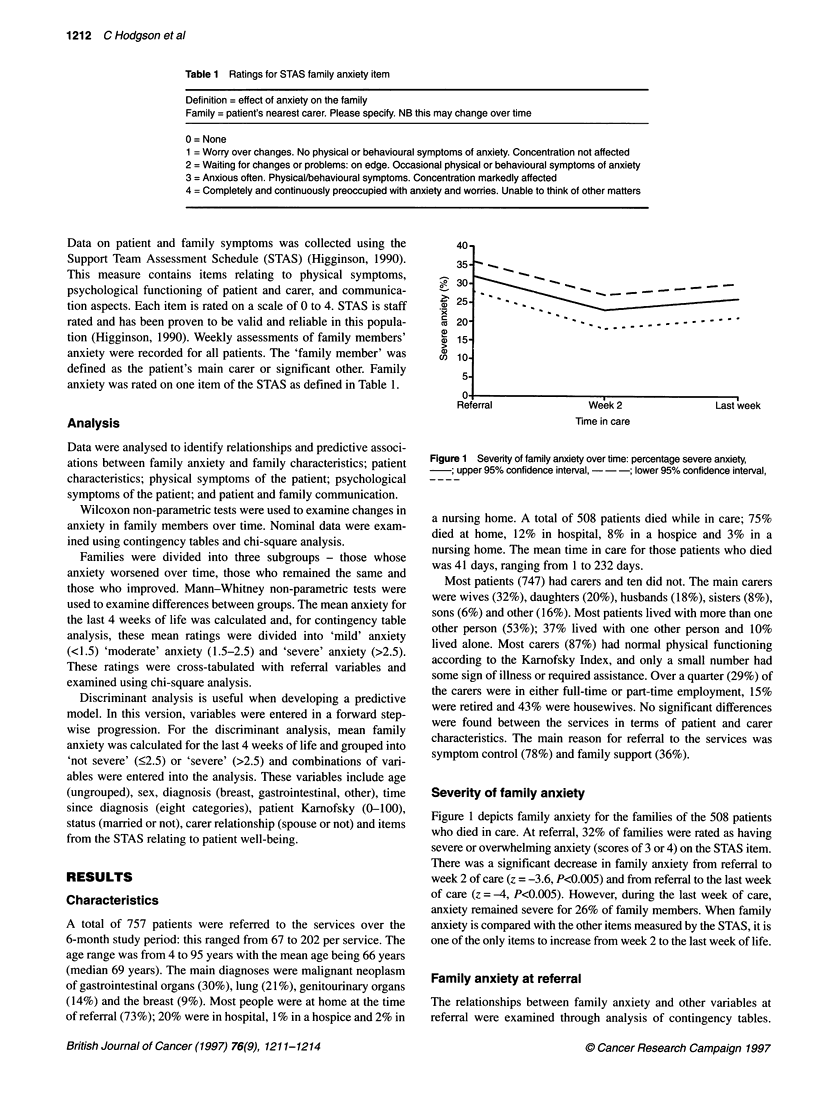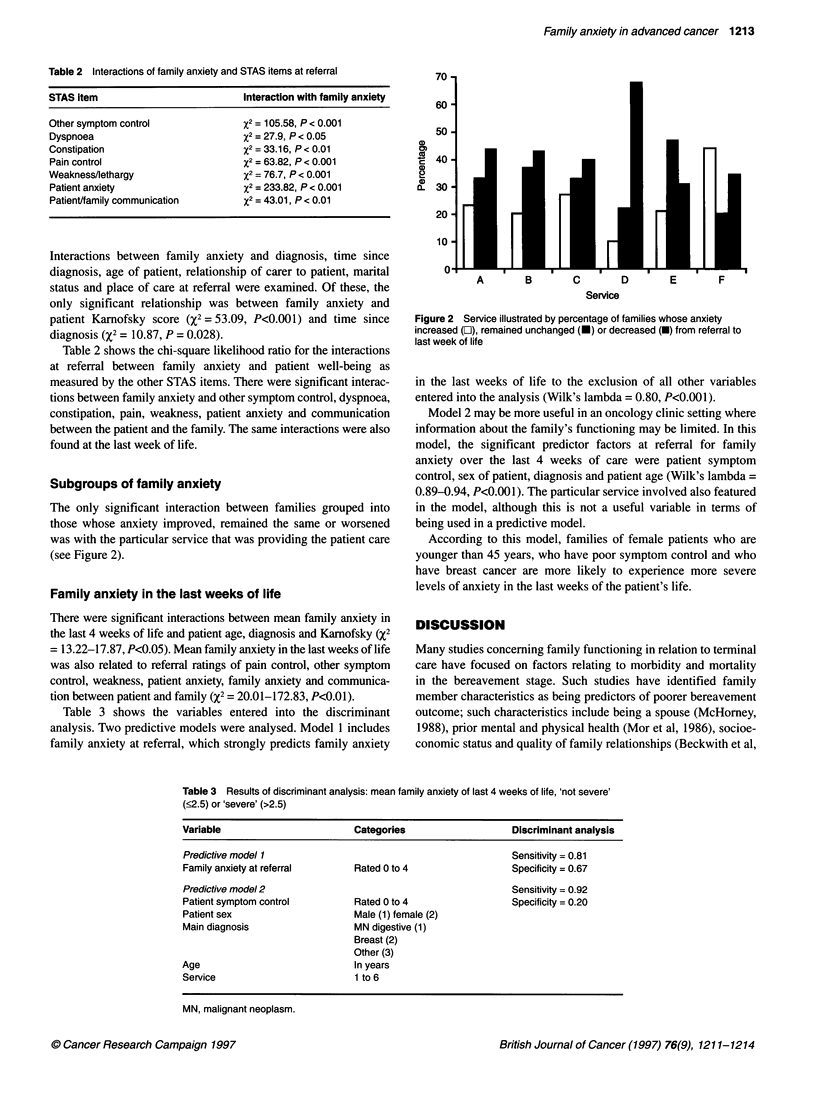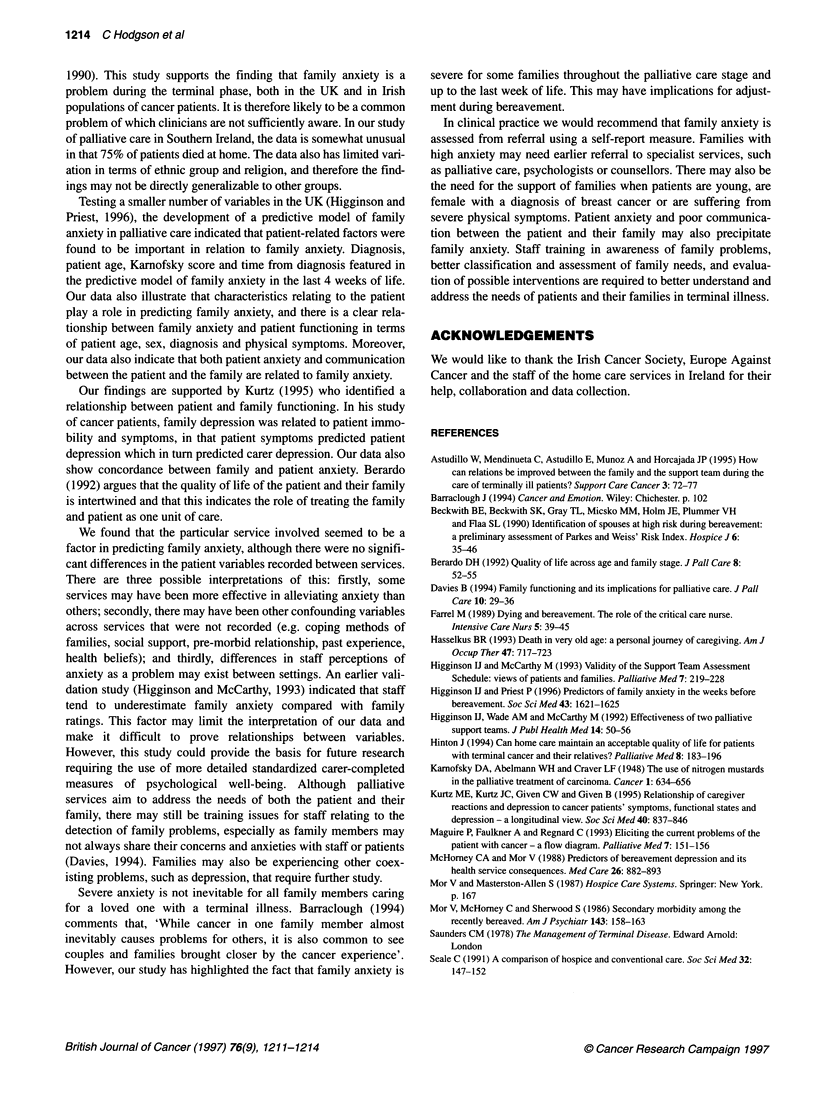# Family anxiety in advanced cancer: a multicentre prospective study in Ireland.

**DOI:** 10.1038/bjc.1997.535

**Published:** 1997

**Authors:** C. Hodgson, I. Higginson, M. McDonnell, E. Butters

**Affiliations:** Department of Palliative Care and Policy, Rayne Institute, Kings College School of Medicine and Dentistry, London, UK.

## Abstract

Six home care services in Southern Ireland collected data on a total of 757 patients over a 6-month period. Patient and family well-being were measured using the staff-rated Support Team Assessment Schedule and Karnofsky Index. Five hundred and eight patients died while in care, 75% of whom died at home. At referral, 32% of families were rated as having severe or overwhelming anxiety. During the last week of care, anxiety remained severe for 26% of family members. Patient and family well-being were inter-related, and there were significant interactions between family anxiety and patient physical and psychological symptoms, and communication. Discriminant analysis produced two predictive models. In model 1, family anxiety at referral strongly predicts family anxiety in the last week of life. In model 2, family anxiety at referral is excluded from the analysis, and the significant predictor factors at referral for family anxiety in the last 4 weeks of life are patient symptom control, sex of patient, diagnosis and patient age.


British Journal of Cancer (1997) 76(9), 1211–1214
© 1997 Cancer Research Campaign

### Family anxiety in advanced cancer: a multicentre prospective study in Ireland

**C Hodgson[1], I Higginson[1], M McDonnell[2] and E Butters[3]**

[1]Department of Palliative Care and Policy, Rayne Institute, Kings College School of Medicine and Dentistry, 123 Coldharbour Lane, London SE5 9NU, UK; [2]St Gabriel's Ward, Our Lady's Hospice, Harold's Cross, Dublin 6, Ireland; [3]Health Services Research Unit, London School of Hygiene and Tropical Medicine, Keppel Street, London WC1E 7HT, UK

**Summary** Six home care services in Southern Ireland collected data on a total of 757 patients over a 6-month period. Patient and family well-being were measured using the staff-rated Support Team Assessment Schedule and Karnofsky Index. Five hundred and eight patients died while in care, 75% of whom died at home. At referral, 32% of families were rated as having severe or overwhelming anxiety. During the last week of care, anxiety remained severe for 26% of family members. Patient and family well-being were inter-related, and there were significant interactions between family anxiety and patient physical and psychological symptoms, and communication. Discriminant analysis produced two predictive models. In model 1, family anxiety at referral strongly predicts family anxiety in the last week of life. In model 2, family anxiety at referral is excluded from the analysis, and the significant predictor factors at referral for family anxiety in the last 4 weeks of life are patient symptom control, sex of patient, diagnosis and patient age.

**Keywords**: family anxiety; palliative care; predictive model

While caring for a dying relative can be a rewarding experience, families vary in their ability to cope, and some inevitably find caring stressful as they are faced with major psychological and physical demands at a time when they may feel less able to cope (Hasselkus, 1993). Palliative care has developed with the aim of providing support to people who are dying and their relatives (Saunders, 1978; Seale, 1991). However, the problems and needs of family members caring for a loved one in the terminal stages of illness have rarely been studied, and most research has focused on families in the bereavement period (Mor and Masterton-Allen, 1987).

Barraclough (1994) has estimated that depression and anxiety are almost as common among spouses as among patients. A prospective study of patients and their families receiving palliative home care services indicated that 17% of relatives were experiencing high levels of depression and 14% were experiencing anxiety in the later stages of care (Hinton, 1994). In five palliative care teams in England, family anxiety was one of the most severe problems at referral and did not significantly improve during care but remained severe for many family members during the last week of life (Higginson et al, 1992). Therefore, family anxiety seems to be an important issue for palliative care.

There seems to be concordance between psychological problems of patients and of their families, although the distress suffered is not always shared between the two in an attempt to protect each other (Astudillo, 1995). An understanding of the inter-relationship between the needs of the patient and of the family would help identify risk factors. Few studies have examined patients and families concurrently. However, a study of cancer patients and their families/carers by Kurtz et al (1995) indicated that patients' symptoms predicted patient depression, which in turn predicted carer depression.

Maguire (1993) suggests that many clinicians are not skilled at recognizing the symptoms of psychological problems, and those that do may not feel that they are able to help, or view the reaction as 'normal'. Anxiety in terminal care is expected, but prolonged severe levels may have longer-term effects on family members. For example, psychological problems at the pre-bereavement phase have been linked with a poorer outcome for relatives during the bereavement period (Mor et al, 1986) and unresolved premorbid difficulties may predispose to a poor or traumatic bereavement process (Farrel, 1989).

Therefore, identifying predictor factors for family anxiety during palliative care may enable clinicians to identify risk factors and find resources to address those risks. This may not only improve the quality of life of patients and carers but also prevent some of the problems family members experience during bereavement. This study aimed to examine the characteristics associated with family anxiety in the weeks before the death of a loved one.

### METHOD

### Settings

Six home care services in Ireland routinely collected data prospectively on all patients referred for care over a 6-month period. The teams were hospice based, provided an advisory role and 24-h access and were multidisciplinary with 53.5 whole-time-equivalent staff.

### Outcome measures

Standardized enhanced clinical records were used to collect demographic data. Family member's functioning and ability to self care was assessed using the Karnofsky Index (Karnofsky et al, 1948).

*Received 25 November 1996*
*Revised 1 April 1997*
*Accepted 18 April 1997*

*Correspondence to:* C Hodgson

**Table 1** Ratings for STAS family anxiety item

| |
|---|
| Definition = effect of anxiety on the family<br>Family = patient's nearest carer. Please specify. NB this may change over time |
| 0 = None<br>1 = Worry over changes. No physical or behavioural symptoms of anxiety. Concentration not affected<br>2 = Waiting for changes or problems: on edge. Occasional physical or behavioural symptoms of anxiety<br>3 = Anxious often. Physical/behavioural symptoms. Concentration markedly affected<br>4 = Completely and continuously preoccupied with anxiety and worries. Unable to think of other matters |

Data on patient and family symptoms was collected using the Support Team Assessment Schedule (STAS) (Higginson, 1990). This measure contains items relating to physical symptoms, psychological functioning of patient and carer, and communication aspects. Each item is rated on a scale of 0 to 4. STAS is staff rated and has been proven to be valid and reliable in this population (Higginson, 1990). Weekly assessments of family members' anxiety were recorded for all patients. The 'family member' was defined as the patient's main carer or significant other. Family anxiety was rated on one item of the STAS as defined in Table 1.

### Analysis

Data were analysed to identify relationships and predictive associations between family anxiety and family characteristics; patient characteristics; physical symptoms of the patient; psychological symptoms of the patient; and patient and family communication.

Wilcoxon non-parametric tests were used to examine changes in anxiety in family members over time. Nominal data were examined using contingency tables and chi-square analysis.

Families were divided into three subgroups – those whose anxiety worsened over time, those who remained the same and those who improved. Mann–Whitney non-parametric tests were used to examine differences between groups. The mean anxiety for the last 4 weeks of life was calculated and, for contingency table analysis, these mean ratings were divided into 'mild' anxiety (<1.5) 'moderate' anxiety (1.5–2.5) and 'severe' anxiety (>2.5). These ratings were cross-tabulated with referral variables and examined using chi-square analysis.

Discriminant analysis is useful when developing a predictive model. In this version, variables were entered in a forward stepwise progression. For the discriminant analysis, mean family anxiety was calculated for the last 4 weeks of life and grouped into 'not severe' (≤2.5) or 'severe' (>2.5) and combinations of variables were entered into the analysis. These variables include age (ungrouped), sex, diagnosis (breast, gastrointestinal, other), time since diagnosis (eight categories), patient Karnofsky (0–100), status (married or not), carer relationship (spouse or not) and items from the STAS relating to patient well-being.

### RESULTS

### Characteristics

A total of 757 patients were referred to the services over the 6-month study period: this ranged from 67 to 202 per service. The age range was from 4 to 95 years with the mean age being 66 years (median 69 years). The main diagnoses were malignant neoplasm of gastrointestinal organs (30%), lung (21%), genitourinary organs (14%) and the breast (9%). Most people were at home at the time of referral (73%); 20% were in hospital, 1% in a hospice and 2% in a nursing home. A total of 508 patients died while in care; 75% died at home, 12% in hospital, 8% in a hospice and 3% in a nursing home. The mean time in care for those patients who died was 41 days, ranging from 1 to 232 days.

**Figure 1** Severity of family anxiety over time: percentage severe anxiety, ——; upper 95% confidence interval, — — —; lower 95% confidence interval, - - - -

Most patients (747) had carers and ten did not. The main carers were wives (32%), daughters (20%), husbands (18%), sisters (8%), sons (6%) and other (16%). Most patients lived with more than one other person (53%); 37% lived with one other person and 10% lived alone. Most carers (87%) had normal physical functioning according to the Karnofsky Index, and only a small number had some sign of illness or required assistance. Over a quarter (29%) of the carers were in either full-time or part-time employment, 15% were retired and 43% were housewives. No significant differences were found between the services in terms of patient and carer characteristics. The main reason for referral to the services was symptom control (78%) and family support (36%).

### Severity of family anxiety

Figure 1 depicts family anxiety for the families of the 508 patients who died in care. At referral, 32% of families were rated as having severe or overwhelming anxiety (scores of 3 or 4) on the STAS item. There was a significant decrease in family anxiety from referral to week 2 of care ($z = -3.6$, $P<0.005$) and from referral to the last week of care ($z = -4$, $P<0.005$). However, during the last week of care, anxiety remained severe for 26% of family members. When family anxiety is compared with the other items measured by the STAS, it is one of the only items to increase from week 2 to the last week of life.

### Family anxiety at referral

The relationships between family anxiety and other variables at referral were examined through analysis of contingency tables.

© Cancer Research Campaign 1997

**Table 2** Interactions of family anxiety and STAS items at referral

| STAS item | Interaction with family anxiety |
|---|---|
| Other symptom control | $\chi^2 = 105.58$, $P < 0.001$ |
| Dyspnoea | $\chi^2 = 27.9$, $P < 0.05$ |
| Constipation | $\chi^2 = 33.16$, $P < 0.01$ |
| Pain control | $\chi^2 = 63.82$, $P < 0.001$ |
| Weakness/lethargy | $\chi^2 = 76.7$, $P < 0.001$ |
| Patient anxiety | $\chi^2 = 233.82$, $P < 0.001$ |
| Patient/family communication | $\chi^2 = 43.01$, $P < 0.01$ |

Interactions between family anxiety and diagnosis, time since diagnosis, age of patient, relationship of carer to patient, marital status and place of care at referral were examined. Of these, the only significant relationship was between family anxiety and patient Karnofsky score ($\chi^2 = 53.09$, $P<0.001$) and time since diagnosis ($\chi^2 = 10.87$, $P = 0.028$).

Table 2 shows the chi-square likelihood ratio for the interactions at referral between family anxiety and patient well-being as measured by the other STAS items. There were significant interactions between family anxiety and other symptom control, dyspnoea, constipation, pain, weakness, patient anxiety and communication between the patient and the family. The same interactions were also found at the last week of life.

### Subgroups of family anxiety

The only significant interaction between families grouped into those whose anxiety improved, remained the same or worsened was with the particular service that was providing the patient care (see Figure 2).

### Family anxiety in the last weeks of life

There were significant interactions between mean family anxiety in the last 4 weeks of life and patient age, diagnosis and Karnofsky ($\chi^2 = 13.22$–$17.87$, $P<0.05$). Mean family anxiety in the last weeks of life was also related to referral ratings of pain control, other symptom control, weakness, patient anxiety, family anxiety and communication between patient and family ($\chi^2 = 20.01$–$172.83$, $P<0.01$).

Table 3 shows the variables entered into the discriminant analysis. Two predictive models were analysed. Model 1 includes family anxiety at referral, which strongly predicts family anxiety in the last weeks of life to the exclusion of all other variables entered into the analysis (Wilk's lambda = 0.80, $P<0.001$).

**Figure 2** Service illustrated by percentage of families whose anxiety increased (□), remained unchanged (■) or decreased (■) from referral to last week of life

Model 2 may be more useful in an oncology clinic setting where information about the family's functioning may be limited. In this model, the significant predictor factors at referral for family anxiety over the last 4 weeks of care were patient symptom control, sex of patient, diagnosis and patient age (Wilk's lambda = 0.89–0.94, $P<0.001$). The particular service involved also featured in the model, although this is not a useful variable in terms of being used in a predictive model.

According to this model, families of female patients who are younger than 45 years, who have poor symptom control and who have breast cancer are more likely to experience more severe levels of anxiety in the last weeks of the patient's life.

### DISCUSSION

Many studies concerning family functioning in relation to terminal care have focused on factors relating to morbidity and mortality in the bereavement stage. Such studies have identified family member characteristics as being predictors of poorer bereavement outcome; such characteristics include being a spouse (McHorney, 1988), prior mental and physical health (Mor et al, 1986), socioeconomic status and quality of family relationships (Beckwith et al,

**Table 3** Results of discriminant analysis: mean family anxiety of last 4 weeks of life, 'not severe' (≤2.5) or 'severe' (>2.5)

| Variable | Categories | Discriminant analysis |
|---|---|---|
| *Predictive model 1* | | Sensitivity = 0.81 |
| Family anxiety at referral | Rated 0 to 4 | Specificity = 0.67 |
| *Predictive model 2* | | Sensitivity = 0.92 |
| Patient symptom control | Rated 0 to 4 | Specificity = 0.20 |
| Patient sex | Male (1) female (2) | |
| Main diagnosis | MN digestive (1) | |
| | Breast (2) | |
| | Other (3) | |
| Age | In years | |
| Service | 1 to 6 | |

MN, malignant neoplasm.

© Cancer Research Campaign 1997

1990). This study supports the finding that family anxiety is a problem during the terminal phase, both in the UK and in Irish populations of cancer patients. It is therefore likely to be a common problem of which clinicians are not sufficiently aware. In our study of palliative care in Southern Ireland, the data is somewhat unusual in that 75% of patients died at home. The data also has limited variation in terms of ethnic group and religion, and therefore the findings may not be directly generalizable to other groups.

Testing a smaller number of variables in the UK (Higginson and Priest, 1996), the development of a predictive model of family anxiety in palliative care indicated that patient-related factors were found to be important in relation to family anxiety. Diagnosis, patient age, Karnofsky score and time from diagnosis featured in the predictive model of family anxiety in the last 4 weeks of life. Our data also illustrate that characteristics relating to the patient play a role in predicting family anxiety, and there is a clear relationship between family anxiety and patient functioning in terms of patient age, sex, diagnosis and physical symptoms. Moreover, our data also indicate that both patient anxiety and communication between the patient and the family are related to family anxiety.

Our findings are supported by Kurtz (1995) who identified a relationship between patient and family functioning. In his study of cancer patients, family depression was related to patient immobility and symptoms, in that patient symptoms predicted patient depression which in turn predicted carer depression. Our data also show concordance between family and patient anxiety. Berardo (1992) argues that the quality of life of the patient and their family is intertwined and that this indicates the role of treating the family and patient as one unit of care.

We found that the particular service involved seemed to be a factor in predicting family anxiety, although there were no significant differences in the patient variables recorded between services. There are three possible interpretations of this: firstly, some services may have been more effective in alleviating anxiety than others; secondly, there may have been other confounding variables across services that were not recorded (e.g. coping methods of families, social support, pre-morbid relationship, past experience, health beliefs); and thirdly, differences in staff perceptions of anxiety as a problem may exist between settings. An earlier validation study (Higginson and McCarthy, 1993) indicated that staff tend to underestimate family anxiety compared with family ratings. This factor may limit the interpretation of our data and make it difficult to prove relationships between variables. However, this study could provide the basis for future research requiring the use of more detailed standardized carer-completed measures of psychological well-being. Although palliative services aim to address the needs of both the patient and their family, there may still be training issues for staff relating to the detection of family problems, especially as family members may not always share their concerns and anxieties with staff or patients (Davies, 1994). Families may also be experiencing other coexisting problems, such as depression, that require further study.

Severe anxiety is not inevitable for all family members caring for a loved one with a terminal illness. Barraclough (1994) comments that, 'While cancer in one family member almost inevitably causes problems for others, it is also common to see couples and families brought closer by the cancer experience'. However, our study has highlighted the fact that family anxiety is severe for some families throughout the palliative care stage and up to the last week of life. This may have implications for adjustment during bereavement.

In clinical practice we would recommend that family anxiety is assessed from referral using a self-report measure. Families with high anxiety may need earlier referral to specialist services, such as palliative care, psychologists or counsellors. There may also be the need for the support of families when patients are young, are female with a diagnosis of breast cancer or are suffering from severe physical symptoms. Patient anxiety and poor communication between the patient and their family may also precipitate family anxiety. Staff training in awareness of family problems, better classification and assessment of family needs, and evaluation of possible interventions are required to better understand and address the needs of patients and their families in terminal illness.

### ACKNOWLEDGEMENTS

We would like to thank the Irish Cancer Society, Europe Against Cancer and the staff of the home care services in Ireland for their help, collaboration and data collection.

### REFERENCES

Astudillo W, Mendinueta C, Astudillo E, Munoz A and Horcajada JP (1995) How can relations be improved between the family and the support team during the care of terminally ill patients? *Support Care Cancer* **3**: 72–77

Barraclough J (1994) *Cancer and Emotion.* Wiley: Chichester. p. 102

Beckwith BE, Beckwith SK, Gray TL, Micsko MM, Holm JE, Plummer VH and Flaa SL (1990) Identification of spouses at high risk during bereavement: a preliminary assessment of Parkes and Weiss' Risk Index. *Hospice J* **6**: 35–46

Berardo DH (1992) Quality of life across age and family stage. *J Pall Care* **8**: 52–55

Davies B (1994) Family functioning and its implications for palliative care. *J Pall Care* **10**: 29–36

Farrel M (1989) Dying and bereavement. The role of the critical care nurse. *Intensive Care Nurs* **5**: 39–45

Hasselkus BR (1993) Death in very old age: a personal journey of caregiving. *Am J Occup Ther* **47**: 717–723

Higginson IJ and McCarthy M (1993) Validity of the Support Team Assessment Schedule: views of patients and families. *Palliative Med* **7**: 219–228

Higginson IJ and Priest P (1996) Predictors of family anxiety in the weeks before bereavement. *Soc Sci Med* **43**: 1621–1625

Higginson IJ, Wade AM and McCarthy M (1992) Effectiveness of two palliative support teams. *J Publ Health Med* **14**: 50–56

Hinton J (1994) Can home care maintain an acceptable quality of life for patients with terminal cancer and their relatives? *Palliative Med* **8**: 183–196

Karnofsky DA, Abelmann WH and Craver LF (1948) The use of nitrogen mustards in the palliative treatment of carcinoma. *Cancer* **1**: 634–656

Kurtz ME, Kurtz JC, Given CW and Given B (1995) Relationship of caregiver reactions and depression to cancer patients' symptoms, functional states and depression – a longitudinal view. *Soc Sci Med* **40**: 837–846

Maguire P, Faulkner A and Regnard C (1993) Eliciting the current problems of the patient with cancer – a flow diagram. *Palliative Med* **7**: 151–156

McHorney CA and Mor V (1988) Predictors of bereavement depression and its health service consequences. *Med Care* **26**: 882–893

Mor V and Masterson-Allen S (1987) *Hospice Care Systems.* Springer: New York. p. 167

Mor V, McHorney C and Sherwood S (1986) Secondary morbidity among the recently bereaved. *Am J Psychiatr* **143**: 158–163

Saunders CM (1978) *The Management of Terminal Disease.* Edward Arnold: London

Seale C (1991) A comparison of hospice and conventional care. *Soc Sci Med* **32**: 147–152

© Cancer Research Campaign 1997